# Polymerase-amplified release of ATP (POLARA) for detecting single nucleotide variants in RNA and DNA†
†Electronic supplementary information (ESI) available. See DOI: 10.1039/c8sc03901a


**DOI:** 10.1039/c8sc03901a

**Published:** 2019-01-30

**Authors:** Michael G. Mohsen, Debin Ji, Eric T. Kool

**Affiliations:** a Department of Chemistry , Stanford University , Stanford , CA 94305 , USA . Email: kool@stanford.edu

## Abstract

The identification of single nucleotide polymorphisms (SNP) is increasingly important for diagnosis and treatment of disease. Here we studied the potential use of ATP-releasing nucleotides (ARNs) for identifying SNPs in DNA and RNA targets. Synthesized as derivatives of the four canonical deoxynucleotides, ARNs can be used in the place of deoxynucleoside triphosphates to elongate a primer hybridized to a nucleic acid template, with the leaving group being ATP rather than pyrophosphate. The released ATP is then harnessed in conjunction with luciferase to generate chemiluminescence. Extension on a long target DNA or RNA generates many equivalents of ATP per target strand, providing isothermal amplification of signal. In principle, allele-specific primers could be used in conjunction with ARNs to generate differential luminescence signals with respect to distinct genetic polymorphisms. To test this, varied primer designs, modifications, enzymes and conditions were tested, resulting in an optimized strategy that discriminates between differing nucleic acid templates with single nucleotide resolution. This strategy was then applied to diagnostically relevant alleles resulting in discrimination between known polymorphisms. SNP detection was successfully performed on transcribed mRNA fragments from four different alleles derived from *JAK2*, *BCR-ABL1*, *BRAF*, and *HBB*. To investigate background interference, wild-type and mutant transcripts of these four alleles were tested and found to be easily distinguishable amid total cellular RNA isolated from human blood. Thus, ARNs have been employed with specialized allele-specific primers to detect diagnostically important SNPs in a novel method that is sensitive, rapid, and isothermal.

## Abstract 2

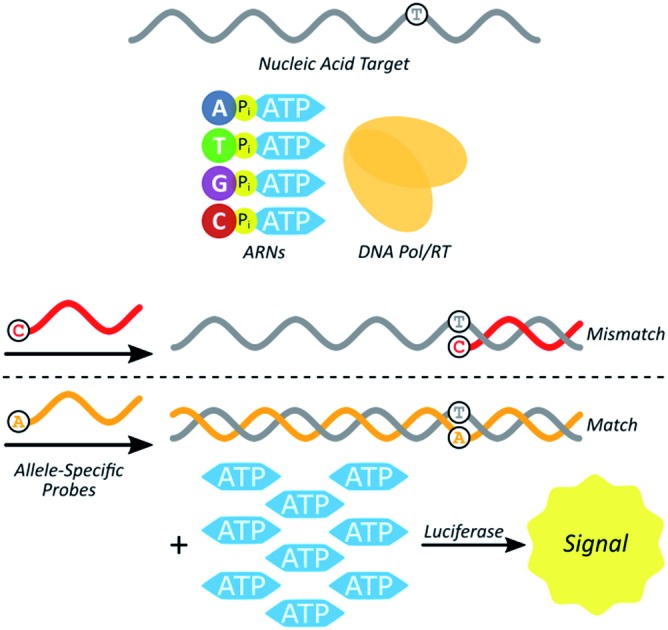
ATP-releasing nucleotides are employed to detect single nucleotide polymorphisms in a novel method that is sensitive, rapid, and isothermal.

## Introduction

At the nucleotide level, genetic variation among all human individuals is estimated to be as low as 0.1–0.5%.^1^,^2^ However, given a genome size of 3.2 billion base pairs, this implies that several million bases differ from person to person. The vast majority of these disparities occur as single base substitutions, known as single nucleotide polymorphisms (SNP).^3^ By definition, SNPs are known substitutions occurring at specific genomic locations and they are becoming increasingly important as genetic markers.^3^,^4^ Most SNPs are of little biological consequence, and of those that are biologically functional, the majority are benign and can account for variations as innocuous as eye color.^3^ There is, however, a smaller subset of SNPs that are considered risk-associated alleles, which are linked to diseases.^3^

Examples of medically relevant SNPs include *JAK2* V617F (1849 G → T), *BCR-ABL1* T315I (944 C → T), *BRAF* V600E (1799 T → A), and *HBB* V6E (20 A → T). In the case of the *JAK2* V617F polymorphism, a change in the amino acid sequence from valine to phenylalanine causes Janus kinase 2 to be conformationally compromised.^5^ Patients who have this mutation suffer from polycythemia vera (PV), a form of myeloproliferative disease.^5^ This SNP occurs in approximately 95% of patients who suffer from PV, so it is imperative for physicians to identify patients who have the V617F mutation.^5^ The T315I mutation in the *BCR-ABL1* fusion gene is known to provide resistance to Gleevec (Imanitib), a small-molecule inhibitor of leukemia-initiating *BCR-ABL* tyrosine kinase.^6^ Imatinib is the therapeutic standard for patients diagnosed with chronic myeloid leukemia (CML), but approximately one-third of CML patients have the *BCR-ABL1* T315I mutation and are consequently resistant to this treatment.^6^ The *BRAF* V600E mutation is linked to a host of cancers including melanoma, non-small cell lung cancer, colorectal cancer, and thyroid cancer.^7^ The mutation from valine to glutamic acid at this position results in enhanced *BRAF* kinase activity and increased phosphorylation of downstream targets.^8^ Vemurafenib (Zelboraf) is a *BRAF* kinase inhibitor administered to cancer patients who test positive for the V600E mutation.^9^ Finally, the V6E mutation in the *HBB* gene is established as the cause of 60–70% of sickle cell disease cases in the United States.^10^ For all of these alleles and many others as well, the ability to detect SNPs is essential because the genetic differences affect disease diagnosis, prognosis, and treatment.

A rapid and accurate method for detecting SNPs is important in informing prognoses and treatments for patients who possess these genetic variations. For example, *BRAF*-mutated melanoma is known to manifest more aggressively in patients than *BRAF* wild-type melanoma.^11^*BRAF*-mutant tumors are more likely to metastasize to the brain than *BRAF* wild-type tumors, and are also linked to decreased likelihood of survival in patients with stage IV cancer.^12^ It is thus crucial to quickly determine whether melanoma patients have *BRAF*-mutated tumors to select the appropriate treatment.

Recent advances in high-throughput sequencing have significantly reduced costs of whole-genome analysis,^13^,^14^ but these techniques remain prohibitively expensive, require specialized equipment, and provide much more data than needed for SNP analysis.^15^ The most common genotyping method for SNP analysis is allele-specific PCR, which is more practical for assaying SNPs in a laboratory setting.^16^–^19^ The basis of allele-specific PCR involves thermostable polymerase extension of a primer only when the primer's 3′ end is perfectly complementary to the template.^20^ Discrimination is achieved by extension when the SNP is complementary to the primer at the 3′-terminal nucleotide, and inhibited extension in the case of a mismatch. However, allele-specific PCR requires thermal cycling equipment and necessitates the use of gel electrophoresis or real-time PCR technology for data readout.^21^ Because PCR is a sensitive method with risks of contamination and artifacts, it is carried out at specialized facilities separate from clinics, requiring added time and increasing costs. The often-urgent nature of medical diagnostic decision-making places value on more rapid techniques that may be less specialized and sensitive to conditions and contamination, and some point-of-care applications (such as in economically limited environments) may find it difficult to obtain real-time PCR equipment.

There are several existing methods for SNP detection that are isothermal and so do not require a thermal cycler.^22^ Pyrosequencing involves a sequence-by-synthesis approach in which nucleotide triphosphates (dNTPs) are added one at a time, and the released PP_i_ is used to generate chemiluminescence through a cascade of enzymatic reactions.^23^,^24^ Padlock probe-initiated rolling circle amplification (RCA) uses the target nucleic acid as a ligation template, and can discriminate between targets at single nucleotide resolution.^25^,^26^ The Invader® assay utilizes an invader probe complementary to the polymorphic site, and two allele-specific probes complementary to either polymorphism with an overhang at their 5′ ends.^27^,^28^ When hybridized to a target, the invader and matched allele-specific probe form a structure recognized and cleaved by a flap endonuclease enzyme, causing a fluorescence signal.^27^,^28^ However, these methods are either expensive or tedious, require specialized technical experience to perform, and may not be readily available in a clinical laboratory setting.

**Fig. 1:**
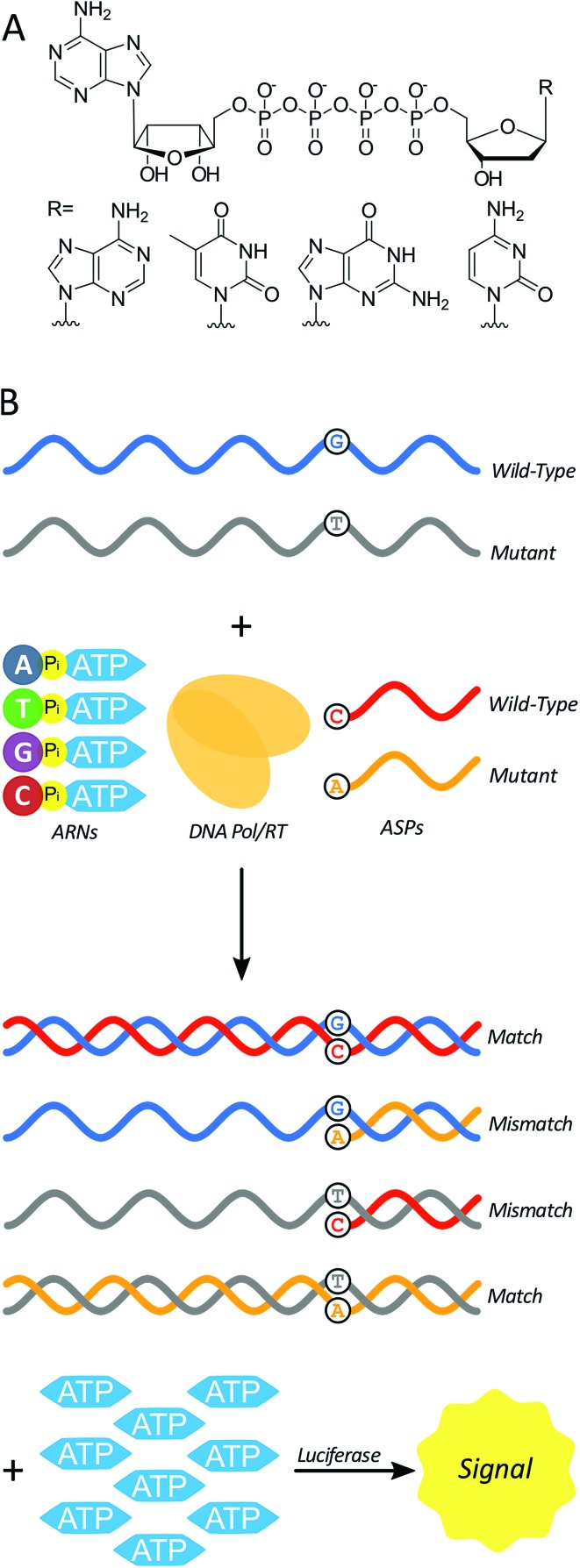
(A) The four chimeric ATP-releasing nucleotide (ARN) analogs of canonical deoxynucleoside triphosphates: dAp_4_A, dTp_4_A, dGp_4_A, and dCp_4_A. (B) Using ARNs in a strategy to differentiate between wild-type and mutant single-stranded nucleic acid (*e.g.* mRNA) sequences. Allele-specific probes (ASPs), ARNs, and a DNA polymerase or reverse transcriptase enzyme react with a single-stranded template to yield a double-stranded product in the case of a match, and inhibited reaction in the case of a mismatch. The production of double-stranded nucleic acid generates multiple equivalents of ATP as a by-product, which is consumed by luciferase to produce a luminescence signal.

Here we test a new isothermal approach for amplified detection of SNPs. In this approach, luminescence signals are generated by release of ATP from chimeric nucleotides (ATP-releasing nucleotides (ARNs, Fig. 1A)) during polymerase extension of primers. For SNP discrimination, allele-specific oligonucleotide primers are used to discriminate between single-stranded nucleic acid targets differing by a single nucleotide. In the case of a match between an allele-specific probe (ASP) oligonucleotide and the target, extension of the probe to form double-stranded nucleic acid material also yields ATP as a by-product (Fig. 1B). In previous studies, polymerase with 3′ → 5′ exonuclease activity digested the primer starting from the 3′ end.^29^ As a result, allele-specific extension was not possible since the ASP's 3′ end contains the allele-specific residue. However, a polymerase lacking 3′ → 5′ exonuclease activity (exo-) should allow for primer extension without altering the ASP, and thus the possibility of single-nucleotide discrimination. The amount of ATP produced is stoichiometrically large, because there is one ATP molecule released for each ARN that is incorporated into the growing strand by polymerase. The produced ATP can then be quantified using a luciferase-based assay to generate a luminescence signal. In principle, this approach could be used to distinguish SNPs in DNAs (using a DNA polymerase) or in RNAs (using a reverse transcriptase). The proposed polymerase-amplified release of ATP (POLARA) method to detect SNPs is isothermal, time-efficient, simple in practice, and highly sensitive due to the large amount of signal generated from priming long genetic targets.

## Results

In order to determine the feasibility of the POLARA method for SNP discrimination, we first constructed a model system using 50 nt synthetic single-stranded DNA targets. The sequences were excerpts from the four aforementioned genes (*BRAF*, *JAK2*, *BCR-ABL1*, *HBB*) containing the polymorphic site at the 18^th^ nucleotide from the 5′ end. Corresponding 18mer synthetic allele-specific probe (ASP) oligonucleotides were designed to have the 3′-terminal base complementary to either the wild-type or mutant SNP. In total, 8 DNA targets and 8 ASPs were used, corresponding to the wild-type or mutant SNP for each allele. The components of a reaction included equimolar DNA target and ASP, the four ARNs, and Klenow exo- DNA polymerase.

The initial results showed that in the case of a mismatch at the SNP site, the DNA polymerase does not extend the ASP, or extends it to a far lesser degree. Using a commercial luciferase-based assay to quantify the relative amounts of ATP in solution, luminescence signals ranged from 4.0-fold to 5.5-fold higher when ASPs and target DNA sequences were correctly matched, while mismatches generated signals that were only slightly above background luminescence (Fig. S1A†). As discussed in a previous study,^29^ background luminescence primarily arises from trace ATP quantities that could not be completely eliminated from ARNs during purification. After subtracting background luminescence from all luminescence signals, the average signal for matches between ASP and target was 29-fold greater than that of mismatches for *JAK2* targets (Fig. S1B†). Clear discrimination between SNPs was achieved with 50mers corresponding to all four alleles. To test whether this approach would be suitable for long targets, two 500 nt single-stranded DNAs corresponding to wild-type and mutant *BRAF* sequences were generated by PCR and strand separation (see ESI for details†). Under similar reaction conditions that succeeded for short targets, wild-type and mutant 500 nt single-stranded *BRAF* DNA targets could also be distinguished using this approach, yielding a 10-fold increase in signal for matches over mismatches after subtracting background luminescence (Fig. S2A†). Though significantly longer, signals generated from 500 nt targets were approximately the same magnitude as those of 50 nt targets (Fig. S2B†). This could be a result of secondary structure in the long single-stranded DNAs, which could impede polymerase from extending the ASP fully. The match-to-mismatch signal ratio for longer targets decreased somewhat relative to that of 50 nt targets, possibly due to mispriming events on the longer, more complex sequences.

After success with DNA targets, long single-stranded RNA targets corresponding to fragments of the allele mRNAs (87–500 nt in length) were transcribed from appropriate DNAs containing T7 RNA polymerase promoters as a more realistic model system for cellular mRNAs. We generated both wild-type and SNP mutant RNA transcripts for each allele. The resulting synthetic RNAs are human mRNA mimics as they contain identical sequence information, albeit in truncated form. To detect SNPs in the mRNA mimics, the approach was modified from that of DNA targets to use reverse transcriptase (RT) lacking RNase H activity in place of DNA polymerase. Initial experiments with Maxima H Minus RT under similar conditions to the DNA targets resulted in satisfactory discrimination between SNPs for *JAK2* and *BRAF* mRNA mimics (Fig. S4†). However, attaining selectivity for *BCR-ABL1* and *HBB* targets proved unsuccessful. It was hypothesized that secondary structure of long RNAs could interfere with the ability of the ASP to hybridize to the target at the SNP site, limiting ASP extension and thus positive signal. Additionally, since RT only requires a few hybridized base pairs to initiate DNA synthesis, even transient nonspecific hybridization of the ASP 3′ end elsewhere on the target RNA could result in nondiscriminatory signals.

To address these issues, the reaction conditions and ASP designs were re-examined in order to increase specificity. Firstly, dCp_4_A was replaced with dCTP in order to improve signal to noise ratios as described in a previous study.^29^ By varying incubation time with working targets, it was found that optimal selectivity for matches over mismatches was observed after 30 minutes (Fig. S5†). Modulating the incubation temperature of the reaction yielded increases in signal as the temperature approached 55 °C, although selectivity suffered (Fig. S6†). This trade-off is likely due to the optimal temperature of Maxima H Minus RT (55 °C) being higher than the ASP melting temperature (*T*_m_) (45 °C on average). Thus 37 °C was selected as the temperature for the reaction. Pre-incubating the reaction mixture at room temperature for 0–90 minutes to allow for slow hybridization had no discernible effect on signal or selectivity (Fig. S7†). Thermal annealing of the ASP on the target also gave no change in selectivity (Fig. S8†). To determine whether accidental 3′-end homology could be a cause of selectivity issues, five different ASPs were used where the first was fully complementary to the target at the polymorphic site, and the other four had the last 6 nucleotides at the 3′ end conserved, as this sequence was repeated a few times throughout the target, and the remaining 12 nucleotides randomly selected using a random number generator. The fully complementary ASP provided nondiscriminatory signal regardless of match or mismatch, while the scrambled ASPs did not generate any signal over background (Fig. S9†). This provided evidence against the hypothesis that non-specific hybridization was contributing substantially to signal.

**Fig. 2:**
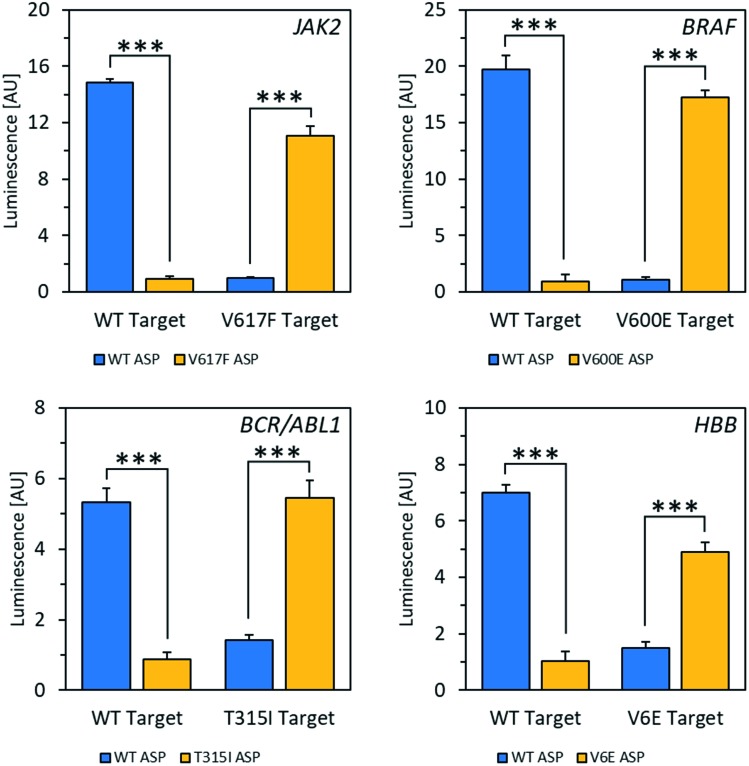
Specificity of wild-type (WT) *vs.* mutant mRNA fragments from four different alleles. RNA targets were detected at concentrations of 100 nM with a 10 times excess of LNA-containing ASPs. Signal increases for matches over mismatches are as follows: *JAK2* (14-fold), *BRAF* (18-fold), *BCR-ABL1* (4.7-fold), *HBB*/beta-globin (4.7-fold). ***P ≤ 0.0001; *t*-test, two-tailed distribution, two sample equal variance. Data are mean ± SD.

Using the mfold web server to predict folding of the target RNAs showed that there was likely an extensive degree of self-complementarity near the polymorphic site.^30^ In order to outcompete secondary structure, locked nucleic acid (LNA)^31^ nucleotides were introduced into the ASPs to increase affinity between probe and target.^32^ Several LNA-containing ASPs were designed with 1–2 LNA nucleotides at or near the 3′ end (Fig. S10†) as well as one with four LNA bases evenly spaced throughout (Fig. S11†). Placing the LNA nucleotides directly at the ASP's 3′ end, which is the most common design used in allele-specific PCR, resulted in decreased specificity (Fig. S10†).^33^ The disparity observed in this case may be because the modification negatively affects RT's ability to differentiate residues with specificity on RNA templates, while allele-specific PCR is typically performed with DNA polymerase on DNA templates. However, including an LNA residue at the second position from the 3′ end enhanced specificity to an acceptable level (Fig. S10†); with 2 LNA nucleotides at the second and third positions from the 3′ end, specificity was improved dramatically. Using the optimized reaction conditions and ASPs containing two LNA nucleotides, excellent selectivity for matches over mismatches was observed in mRNA fragments for all four alleles, with signal increases ranging from 4.3-fold to 18-fold (Fig. 2). Discrimination between wild-type and mutant targets could be reliably determined down to a target RNA concentration of 2.5 nM for *JAK2* and 1 nM for *BRAF* (Fig. S12†). This latter concentration corresponds to 25 femtomoles of target at the test volume. We also tested a strategy using one of the alleles to increase signal for matches over mismatches *via* a double-extension experiment, wherein after 30 minutes of extension by RT, RNase H was used to digest target RNA hybridized to DNA. After this, a primer complementary to the 5′ end of the resulting cDNA was added along with DNA polymerase and incubated for another 30 minutes. For the *JAK2* allele, this resulted in a 29-fold increase in signal for matches over mismatches, improved from 14-fold for a single extension (Fig. S14†). Therefore, this double extension appears to be a viable approach for improving signal over background.

**Fig. 3:**
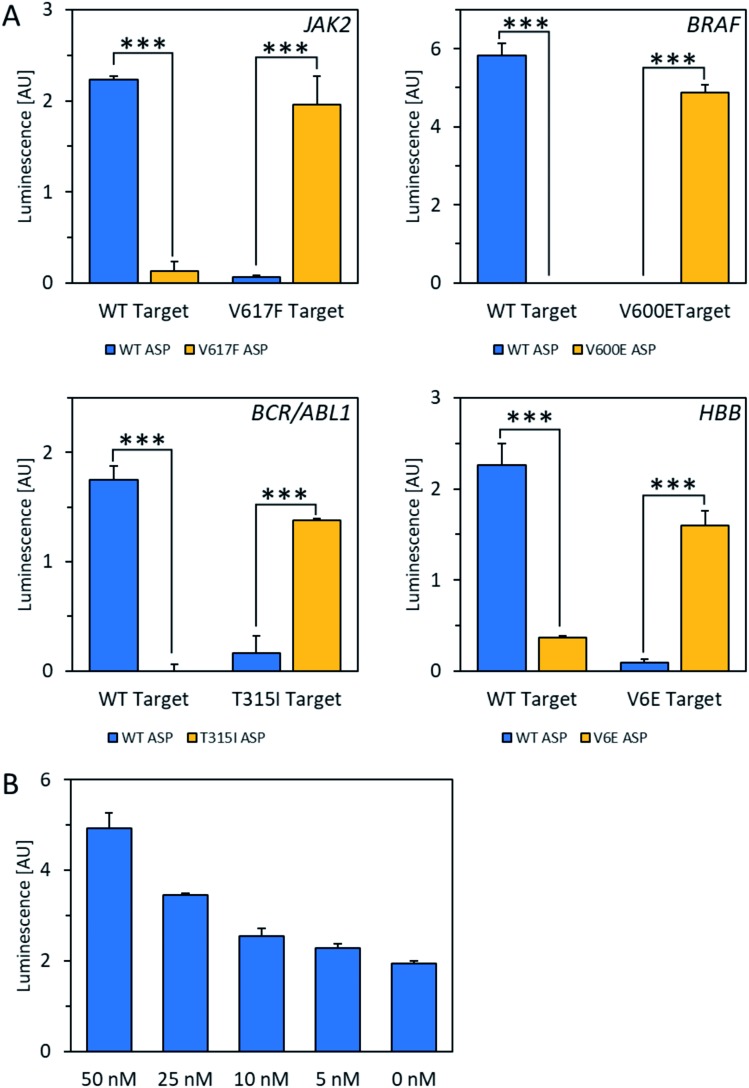
Detection of mRNA fragments amid total cellular RNA isolated from a human blood sample. (A) Specificity of wild-type (WT) *vs.* mutant mRNA mimics amid total cellular RNA isolated from a human blood sample after subtracting background luminescence. Signal increases for matches over mismatches are as follows: *JAK2* (22-fold), *BRAF* (no signal detected above background), *BCR-ABL1* (20-fold), *HBB* (8.5-fold). ***P < 0.0001; *t*-test, two-tailed distribution, two sample equal variance. Data are mean ± SD. Background luminescence was determined by running a parallel experiment without any ASP present. (B) Wild-type *HBB* mRNA mimics were detected above background amid total cellular RNA down to a concentration of 5 nM.

For the POLARA method to be viable as a diagnostic tool, it should have the capacity to detect the target RNA from a pool of total cellular RNA derived from blood, which could cause background interference. To test this issue, total cellular RNA was extracted from a human blood sample and spiked with wild-type and mutant RNA fragments from each of the four alleles. The data show that in all cases, wild-type and mutant RNAs could clearly be discriminated using the standard single extension technique at a concentration of 50 nM amid total cellular RNA (Fig. 3A). After subtracting background luminescence, signal increases for matches over mismatches ranged from 8.5-fold for *HBB*/beta-globin to 20- to 22-fold for *BCR-ABL1* and *JAK2*. Mismatches for the *BRAF* allele were discriminated with even higher specificity, yielding no signal over background, while the matched primers yielded robust signals. Additionally, in dilution experiments the wild-type *HBB* mRNA fragment could be detected above background amid total cellular RNA down to a concentration of 5 nM of the test allele (Fig. 3B). Thus, we conclude that total cellular RNA from blood does not interfere either with allele discrimination or sensitivity over the concentrations tested.

Taken together, our data show that the POLARA approach offers clear single-nucleotide discriminating ability and succeeds in identifying four clinically important alleles even in the background of total cellular RNA. In comparison, AS-PCR performed by gel electrophoresis intrinsically provides a qualitative readout, where mismatch discrimination is assessed by the presence or absence of a target band.^16^,^34^ More recently, allele-specific real-time PCR (RT-PCR) makes use of fluorescent dyes that generate increasing signal as amplification proceeds.^35^ Amplification is observed in the case of both a match and a mismatch but occurs earlier for matches.^36^ This effect is described quantitatively by measuring the difference in cycle threshold (Δ*C*_T_) for matched *vs.* mismatched primers and targets.^36^ Although Δ*C*_T_ is highly dependent on primer design, allele, and several other factors, a Δ*C*_T_ of *ca.* 5–7 is typical for a single mismatch relative to a perfect match using a primer designed to bind to the polymorphic site at its 3′ end.^36^ A Δ*C*_T_ of 5–7 corresponds to a maximal 32 to 128-fold signal increase assuming that the number of amplicons is doubled with every cycle of PCR. Although such fold changes associated with AS-PCR are greater than that of POLARA for three of the four alleles tested here, the signal increase observed with POLARA is obvious and easy to interpret since both alleles are tested side by side. In the case of the *BRAF* allele tested here, essentially complete specificity was realized, yielding no signal over background for mismatches, and highlighting POLARA's potential as an especially promising method for detecting the V600E mutation.

## Conclusions

Although more studies will be needed to test authentic mRNA targets in a series of clinical samples, these early studies suggest the combination of POLARA and specialized allele-specific primers as a novel approach to genetic diagnosis of SNPs. In principle, this method is highly generalizable, as ASPs can easily be synthesized complementary to any SNP site. It is also low-cost and time-efficient; the entire reaction can be completed in 45–75 minutes and is carried out without thermal cycling. A luminometer is needed to measure luminescence produced, and such equipment is common in biomedical laboratories due to the popularity of luminescent reporter assays. Comparatively, AS-PCR is considerably slower, requiring several hours to complete, and necessitates the use of gel electrophoresis or real-time PCR instrumentation.^21^ The new method has the potential to significantly decrease the time and cost of characterizing SNPs in a clinical setting. Future studies will test this possibility.

## Conflicts of interest

There are no conflicts of interest to declare.

## Supplementary Material

Supplementary information
