# Supplementary material for: Polymerase-amplified release of ATP (POLARA) for detecting single nucleotide variants in RNA and DNA
Source: Chem Sci. 2019 Jan 30;10(11):3264–70. doi: 10.1039/c8sc03901a (PMC6429602; doi:10.1039/c8sc03901a)
Supplement: Supplementary file 1 [file SC-010-C8SC03901A-s001.pdf]

Electronic Supplementary Material (ESI) for Chemical Science.
This journal is © The Royal Society of Chemistry 2019

Supplementary Information

Polymerase-Amplified Release of ATP (POLARA) for Detecting Single Nucleotide Variations in DNA and RNA

*Michael G. Mohsen, Debin Ji, Eric T. Kool**

Department of Chemistry, Stanford University, Stanford, CA 94305

*Author to whom correspondence should be addressed: kool@stanford.edu

Table of Contents

Materials and Methods 2
- Materials 2
- Synthesis of ARNs 2
- Synthesis of 500nt DNA Targets 3
- Synthesis of RNA Targets 3
- 50mer DNA Target SNP Discrimination....................4
- 500mer DNA Target SNP Discrimination....................5
- RNA Target SNP Discrimination....................5
- Background tests from blood 6

Supplemental Figures....................7
- Figure S1....................8
- Figure S2....................9
- Figure S3....................10
- Figure S4....................11
- Figure S5....................12
- Figure S6....................13
- Figure S7....................14
- Figure S8....................15
- Figure S9....................16
- Figure S10....................17
- Figure S11....................18
- Figure S12....................20
- Figure S13....................21
- Figure S14....................22

Sequence Data 24
References 27

Materials and Methods

Materials

All nucleotides (dAMP, dTMP, dGMP, dCMP, ATP disodium salts), carbonyl diimidazole (CDI), Dowex-50W ion exchange resin (hydrogen form) were purchased from Sigma Aldrich. Dimethylformamide (DMF) and methanol were purchased from Acros Organics. Klenow fragment DNA Polymerase exo-, DNase I, RNase A, T7 RNA Polymerase, dNTPs, NTPs were purchased from NEB. Platinum Taq High-Fidelity DNA Polymerase, Maxima H Minus Reverse Transcriptase were purchased from Fisher. All buffers used for enzymatic experiments were provided with the enzyme. Plasmids were purchased from Addgene. Kinase Glo reagent was purchased from Promega. All unmodified DNA oligonucleotides were purchased from IDT. LNA-containing oligonucleotides were purchased from Exiqon/Qiagen. High-performance liquid chromatography (HPLC) was performed using a system comprised of 2 Shimadzu LC-10AD pumps, SCL-10A controller, SPD-M10A photodiode array detector. The luminometer used in this experiment was Thermo Fluoroskan Ascent FL. The thermal cycler used for PCR was Eppendorf Mastercycler Gradient.

Synthesis of ARNs

ARNs were synthesized as previously reported.[1] Briefly, the sodium salt of a deoxynucleoside monophosphate (dAMP, dTMP, dGMP, dCMP) and the sodium salt of ATP were each dissolved in distilled deionized water and converted into pyridinium salts using a Dowex-50W ion exchange column (pyridinium form), stirred with 5 equivalents of tributylamine in a 25% methanol solution, and lyophilized to white powders. The resulting tributylammonium nucleotide salts were coevaporated with anhydrous DMF and stored under high vacuum overnight before the subsequent coupling reaction.

The tributylammonium salt of ATP was dissolved in 0.5 mL anhydrous DMF. 6 equivalents CDI was dissolved in 0.1 mL anhydrous DMF and added to the reaction vessel containing ATP. The mixture was stirred at room temperature for 6 hr, after which 30 equivalents of methanol was added to quench the reaction. The reaction was stirred for 10 minutes after which all solvents were removed under high vacuum overnight. The residue was redissolved in 0.5 mL anhydrous DMF. The 8-oxo-2'-deoxyguanosine monophosphate tributylammonium salt was dissolved in 0.5

mL anhydrous DMF and anhydrous $MgCl_2$ (3-5 mg) was added. ATP-imidazolide was added to the reaction vessel containing the monophosphate, and the reaction was stirred at room temperature for 72 hours. 50 mM triethylammonium bicarbonate (TEAB) buffer was added to quench the reaction.

ARNs were purified from the reaction mixture by HPLC using a gradient of acetonitrile and TEAB. The fractions containing the ARN were pooled and lyophilized to yield a white powder. In order to convert the ARN triethylammonium salt to a sodium salt, the lyophilized powder was redissolved in 100 µL MeOH. 400 µL 0.75M NaClO4 in acetone was added, and the mixture was centrifuged at 4°C for 2 minutes at max speed. The mixture was decanted and washed with an additional 400 µL acetone and centrifuged at 4°C for 2 minutes at max speed. The tube was then placed on high vacuum for about an hour until completely dry.

Synthesis of 500nt DNA Targets

PCR was performed on two *BRAF* plasmids,[2] one containing a wild-type *BRAF* insert (a gift from Dustin Maly, Addgene plasmid #40775) and the other containing a *V600E*-mutated insert (Addgene plasmid #53156). The reverse PCR primer was 5′-biotinylated so that the antisense strand could be pulled down with streptavidin beads, leaving only the sense strand. The double-stranded DNA amplicons were purified using Thermo Fisher Scientific GeneJET PCR Purification Kit. Afterward streptavidin magnetic beads (New England Biolabs) were resuspended in the original container by vortexing for 10 seconds. Beads were washed with binding/washing buffer (20 mM Tris-HCl pH 7.5, 1 mM EDTA, .5 M NaCl). After removing all buffer, the biotinylated PCR product was added along with binding/washing buffer and the mixture was left on the shaker at room temperature for 20 minutes. All buffer was removed and 150 mM NaOH was added to denature the double-stranded DNA. The mixture was left on the shaker at RT for 20 minutes, after which the buffer containing single-stranded DNA was pipetted into a new tube.

Synthesis of RNA Targets

For the *JAK2* and *BRAF* genes, PCR was performed on plasmids[2–4] containing inserts with the wild-type (*JAK2*: Addgene plasmid #23915) or mutant (*JAK2* V617F: Addgene plasmid #64610)

sequence to generate 500mer double-stranded DNA templates. The forward PCR primers included the T7 promoter sequence so that the resulting DNA templates would be suitable for T7 RNA transcription. A mutant *BCR-ABL1* plasmid was not available, so the mutation was introduced using a plasmid[5] (Addgene plasmid #27481) with a wild-type *BCR-ABL1* insert by including a mismatch in the reverse PCR primer corresponding to the required mutation. Thus, two 484mer double-stranded DNA template were synthesized by PCR corresponding to the wild-type and mutant sequences. Lastly, since the *HBB* E6V mutation occurs close to the 5′ end of the sequence, two synthetic single-stranded DNAs containing 87 bases of genetic sequence were used as templates for PCR.

In all cases, T7 RNA polymerase was used to generate wild-type and mutant RNA transcripts for each allele. Reactions contained 2.5 μg double-stranded PCR amplicon containing T7 promoter sequence, 0.5 mM NTPs, 5 mM DTT, reaction buffer, T7 RNA polymerase, and nuclease-free water to 50 μL. The reaction was incubated at 37°C for 2 hours. Following transcription, DNase reaction buffer, 2 μL DNase (2 U/μL), and nuclease-free water to 100 μL were added to the same tube. After incubating for an additional hour at 37°C, the resulting RNA transcripts were purified by Zymo Research Clean and Concentrator Kit. The RNA product yielded a clear band on gel electrophoresis that was not present after RNase treatment but remained unperturbed by DNase treatment (Figure S3).

<u>50mer DNA Target SNP Discrimination</u>

Reactions were set up in 600 μL Eppendorf tubes containing 250 nM 50mer DNA target, 250 nM 18mer ASP, 20 μM ARNs (dAp_4A, dTp_4A, dGp_4A, dCp_4A), NEBuffer 2, 0.5 μL (5 units/μL) Klenow fragment DNA Polymerase exo-, and nuclease free water to 20 μL. Three replicates were performed for each of the four possible cases: WT Target/WT ASP (Match), WT Target/Mut ASP (Mismatch), Mut Target/WT ASP (Mismatch), Mut Target/Mut ASP (Mismatch). Background reaction contained no DNA. All reaction vessels were incubated at 37°C for 1 hr. After this time a 10 μL aliquot was taken from each reaction and pipetted into a Greiner 384-well plate. 10 μL Kinase Glo reagent was added to each well and luminescence was recorded after 15 minutes.

500mer DNA Target SNP Discrimination

Reactions were set up in 600 µL Eppendorf tubes containing 100 nM 500mer DNA target, 250 nM 18mer ASP, 20 µM ARNs (dAp$_4$A, dTp$_4$A, dGp$_4$A, dCp$_4$A), NEBuffer 2, 0.5 µL (5 units/µL) Klenow fragment DNA Polymerase exo-, and nuclease free water to 20 µL. Three replicates were performed for each of the four possible cases: WT Target/WT ASP (Match), WT Target/Mut ASP (Mismatch), Mut Target/WT ASP (Mismatch), Mut Target/Mut ASP (Mismatch). Background reaction contained target but no ASP. All reaction vessels were incubated at 37°C for 1 hr. After this time a 10 µL aliquot was taken from each reaction and pipetted into a Greiner 384-well plate. 10 µL Kinase Glo reagent was added to each well and luminescence was recorded after 15 minutes.

RNA Target SNP Discrimination

Reactions were set up in 600 µL Eppendorf tubes containing 100 nM RNA target, 1 µM 18mer ASP, 20 µM ARNs (dAp$_4$A, dTp$_4$A, dGp$_4$A) 20 µM dCTP, RT Reaction buffer, 0.5 µL (200 units/µL) Maxima H Minus reverse transcriptase (RT) and nuclease free water to 20 µL. Three replicates were performed for each of the four possible cases: WT Target/WT ASP (Match), WT Target/Mut ASP (Mismatch), Mut Target/WT ASP (Mismatch), Mut Target/Mut ASP (Mismatch). Background reaction contained RNA target but no ASP. All reaction vessels were incubated at 37°C for 30 min, except in the case of the shorter *HBB* targets, which were incubated for 1 hr. After this time a 10 µL aliquot was taken from each reaction and pipetted into a Greiner 384-well plate. 10 µL Kinase Glo reagent was added to each well and luminescence was recorded after 15 minutes.

For double-extension experiment, reaction tubes containing 100 nM *JAK2* mRNA mimics, 1 µM ASP, 20 µM ARNs (A, T, G), 20 µM dCTP, and Maxima H Minus RT were incubated at 37°C for 30 min. After 30 min, RNAse-H was added to each mixture, which was then incubated at 37°C for 15 min. After this time, 0.5 µL Klenow exo- and 2.0 µL 10 µM *JAK2* Forward Primer were added to each reaction vessel, which was then incubated at 37°C for an additional 30 min. After this time a 10 µL aliquot was taken from each reaction and pipetted into a Greiner 384-well plate. 10 µL Kinase Glo reagent was added to each well and luminescence was recorded after 15 minutes.

Background tests from blood

PreAnalytiX PAXgene Blood RNA Kit was used to isolate RNA from human blood. Reactions were set up in 600 µL Eppendorf tubes containing 100 ng blood RNA, 50 nM (165 ng) enriched RNA target, 1 µM 18mer ASP, 20 µM ARNs (dAp_4A, dTp_4A, dGp_4A) 20 µM dCTP, RT Reaction buffer, 0.5 µL (200 units/µL) Maxima H Minus reverse transcriptase (RT) and nuclease free water to 10 µL. For the limit of detection experiment, blood RNA was kept constant at 100 ng and the amount of enriched RNA target was varied from 50 nM to 5 nM. Reaction vessels were incubated for 1 hr at 37°C. After this time a 5 µL aliquot was taken from each reaction and pipetted into a Greiner 384-well plate. 15 µL Kinase Glo reagent was added to each well and luminescence was recorded after 15 minutes.

Supplemental Figures

A.

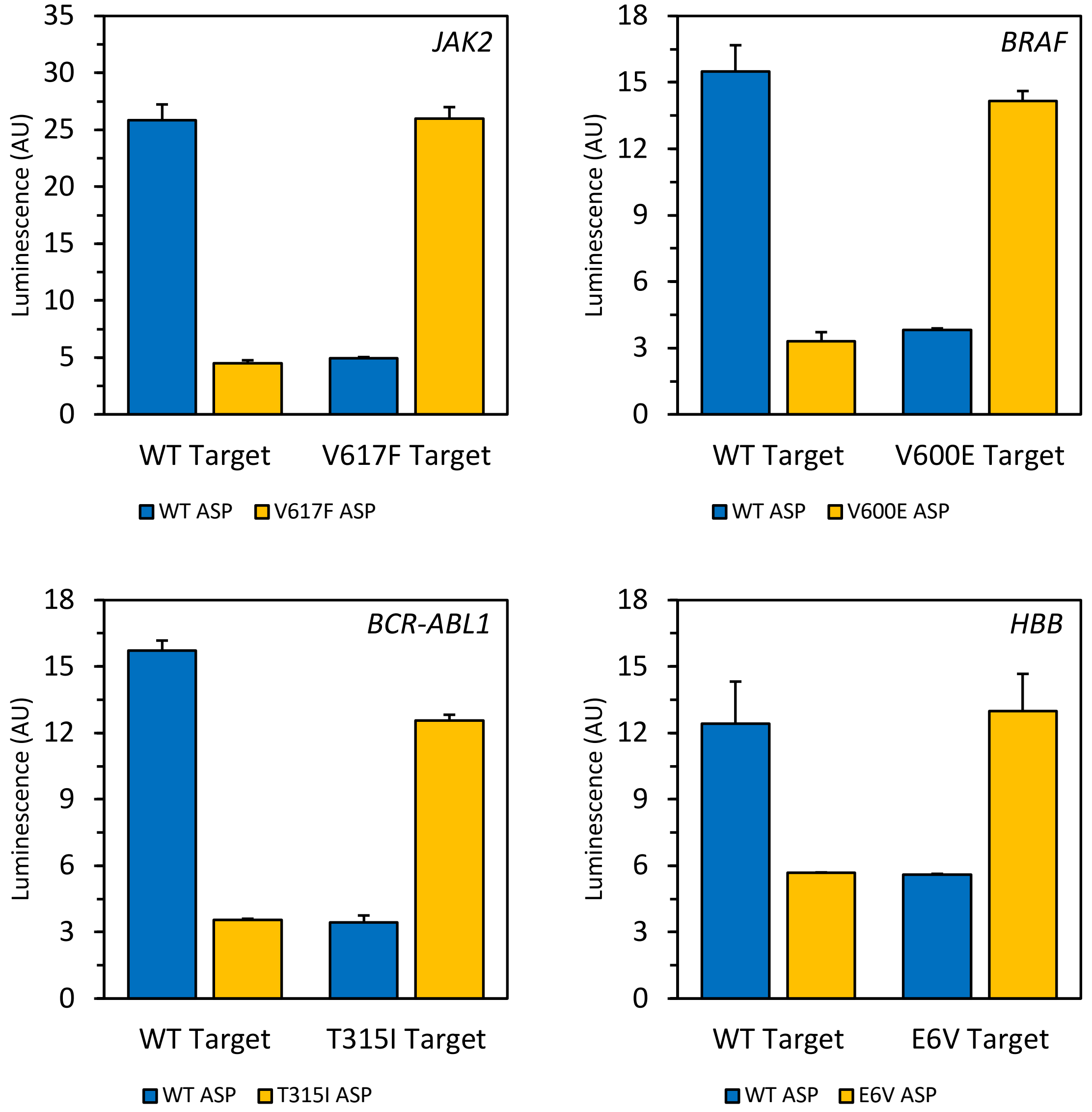

B.

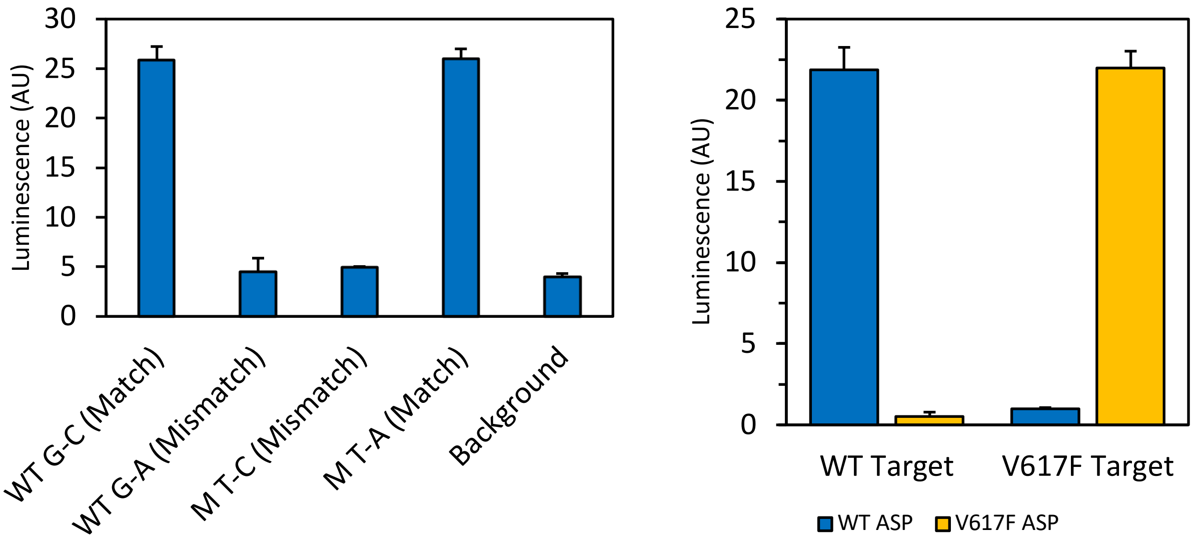

Figure S1. Specificity for matches over mismatches with 50nt synthetic wild-type (WT) and mutant (M) DNA targets. The first letter after WT/M is the base at the polymorphic site on the target, and the second letter is the base at the 3′ end of the ASP. **A.** (250 nM) and 18nt ASPs (250 nM). Treated with 20 µM ARNs and Klenow fragment (Kf) exo- for 1 hr at 37°C. *JAK2*/V617F (5.5-fold increase); *BRAF*/V600E (4.2-fold increase); *BCR-ABL1*/T315I (4.0-fold increase); *HBB*/E6V (2.2-fold increase). **B.** Subtracting background fluorescence from both signals, the signal increase is 29-fold for matches over mismatches in the case of *JAK2* 50mer DNA targets. In the left chart showing background luminescence, “WT” (wild-type) and “M” (mutant; V617F) refer to the target SNP (WT: G, V617F: T), and the base after the hyphen corresponds to the ASP 3′ end (WT: C, V617F: A).

A.

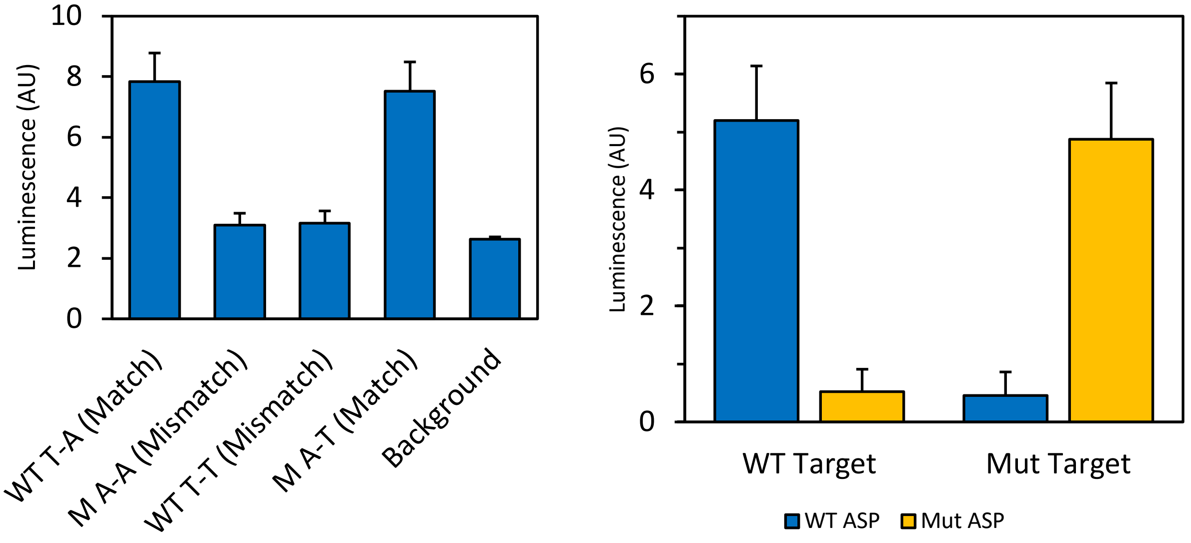

B.

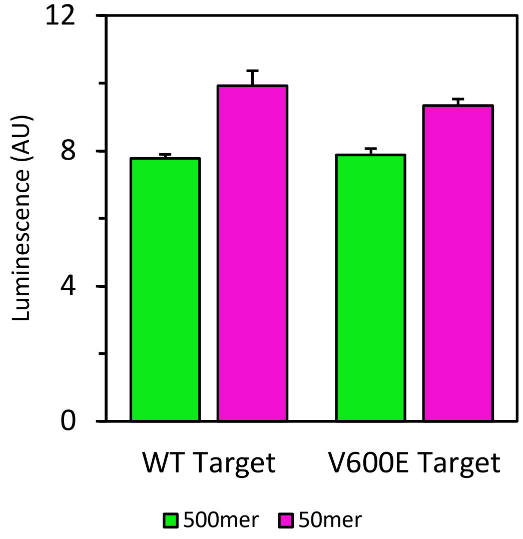

Figure S2. **A.** Specificity for matches over mismatches with 500nt DNA *BRAF* targets (100 nM) and 18nt ASPs (250 nM). Treated with 20 µM ARNs (all four) and Kf exo- for 1 hr at 37°C. Subtracting background fluorescence gives a signal increase of 10-fold for matches over mismatches. **B.** Comparing singles generated from matched targets (100 nM) and ASPs (1 µM) for 500mer targets and 50mer targets. Treated with 20 µM ARNs (all four) and Kf exo- for 1 hr at 37°C. Signals for 50mer matches were slightly higher than those of 500mer matches.

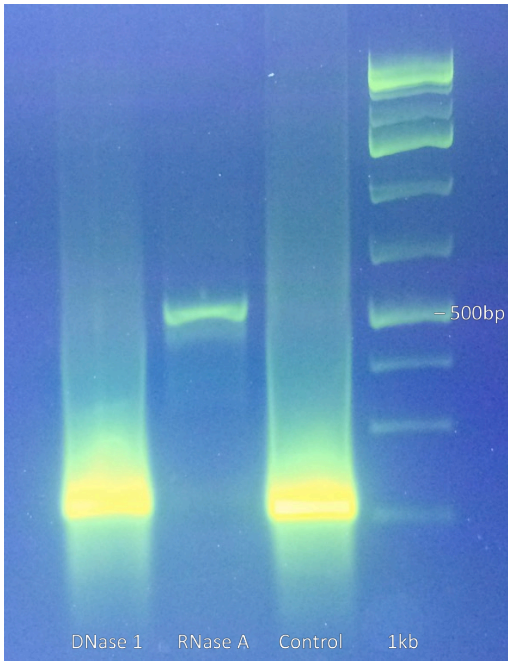

Figure S3. Agarose gel depicting *BRAF* mRNA fragment synthesized by T7 RNA polymerase. The first lane from the left contains the reaction mixture treated with DNase 1, the RNA band is clearly present. The second lane contains the reaction mixture treated with RNase A, and only the 500mer double-stranded DNA template is observed. The third lane contains reaction mixture with no treatment. The fourth lane contains 1KB Plus ladder for reference. 2.5% agarose gel, SYBR Gold dye, run for 50 min at 95 V.

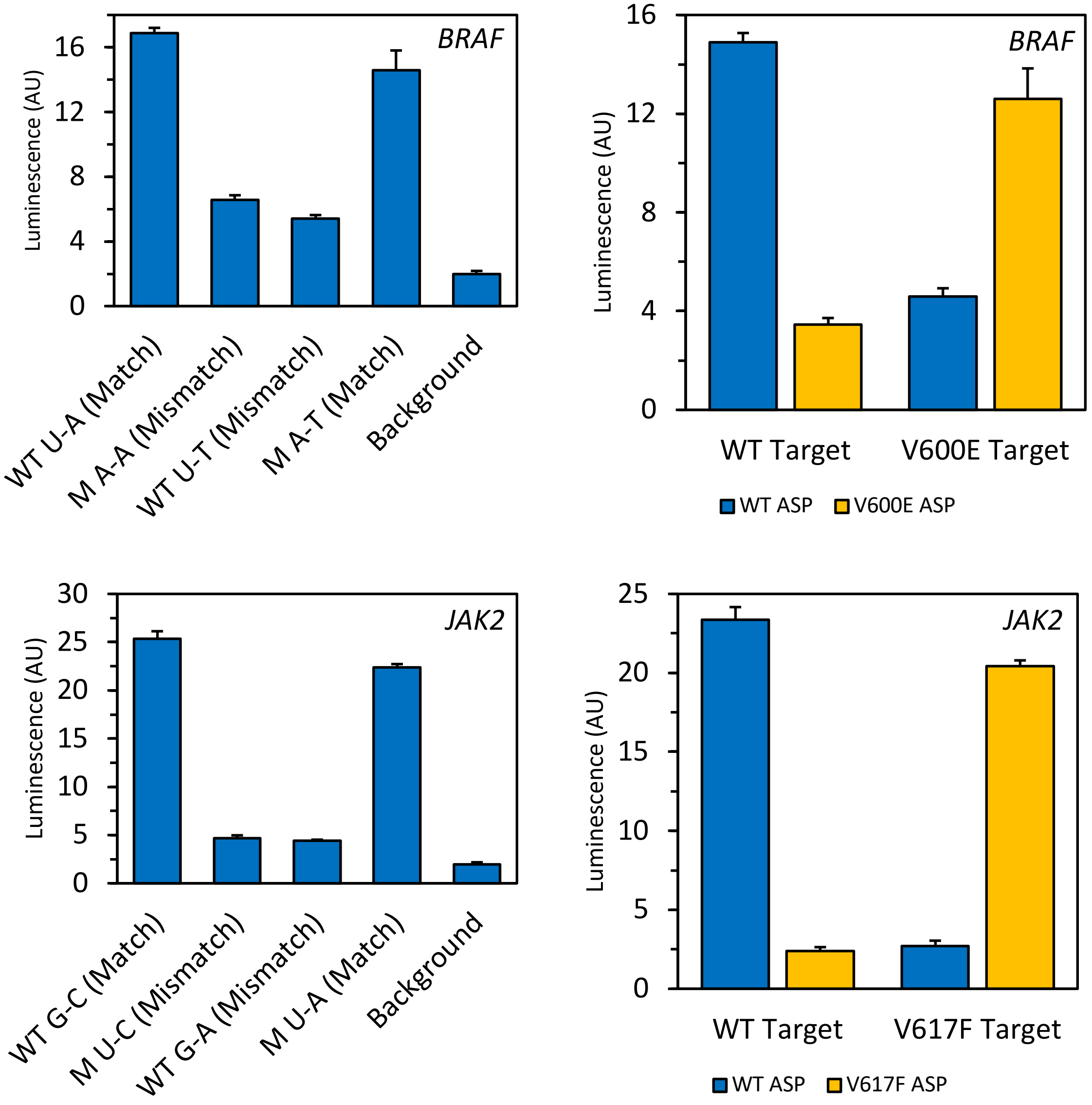

Figure S4. Specificity of POLARA for matches over mismatches with ~500mer RNA targets. 100 nM target, 1 μM ASP, 20 μM ARNs (all four), Maxima H Minus RT, incubated for 30 min at 37°C. After subtracting background signals, 3.4-fold signal increase was observed for *BRAF* RNA targets and 8.5-fold signal increase was observed for *JAK2* RNA targets.

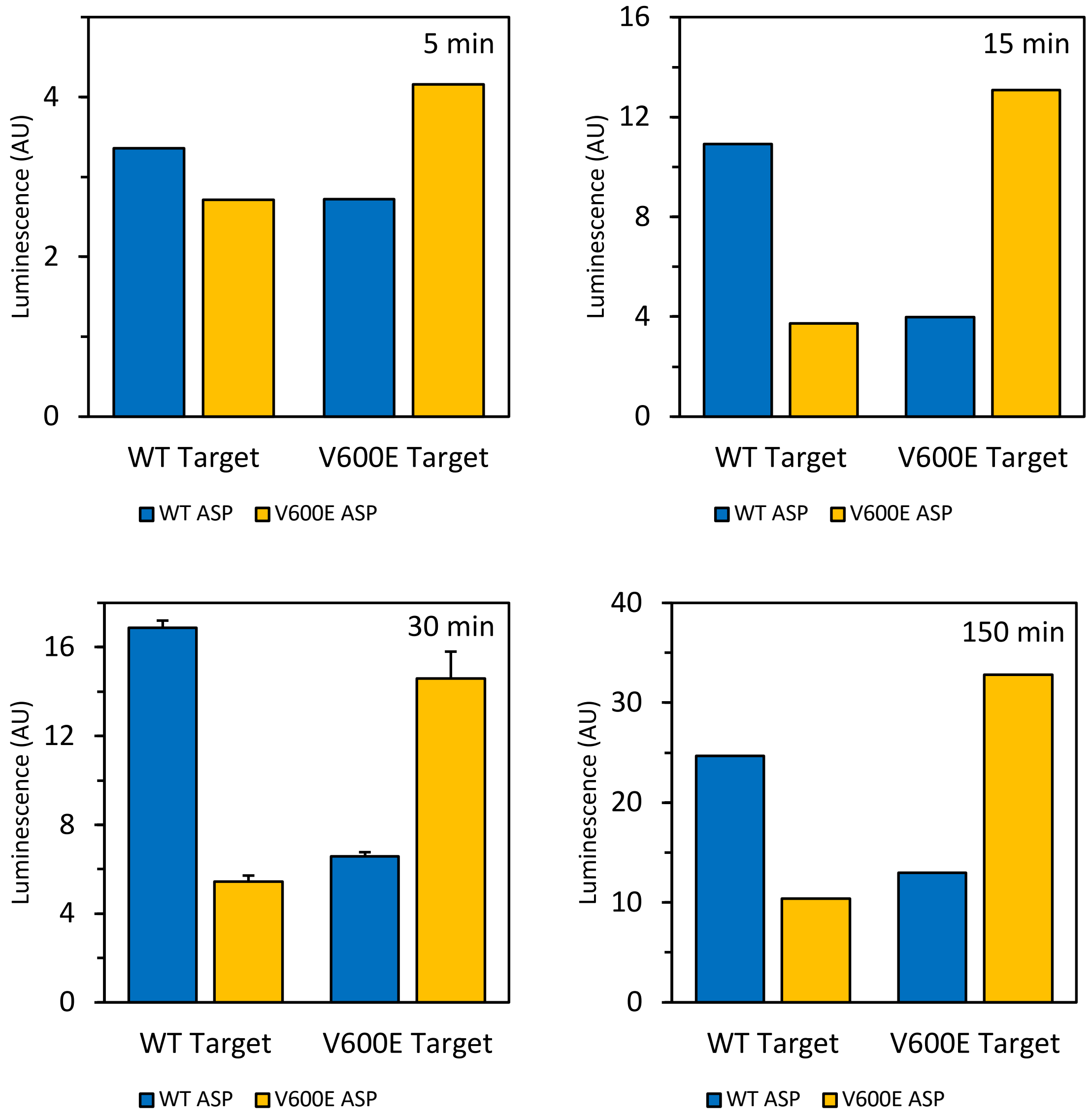

Figure S5. Specificity of POLARA for matches over mismatches with 500mer *BRAF* RNA targets with varied incubation time. 100 nM target, 1 µM ASP, 20 µM ARNs (all four), Maxima H Minus RT, incubated for varying time at 37°C. Increasing the amount of time to 30 minutes increased specificity, but beyond 30 minutes specificity was decreased.

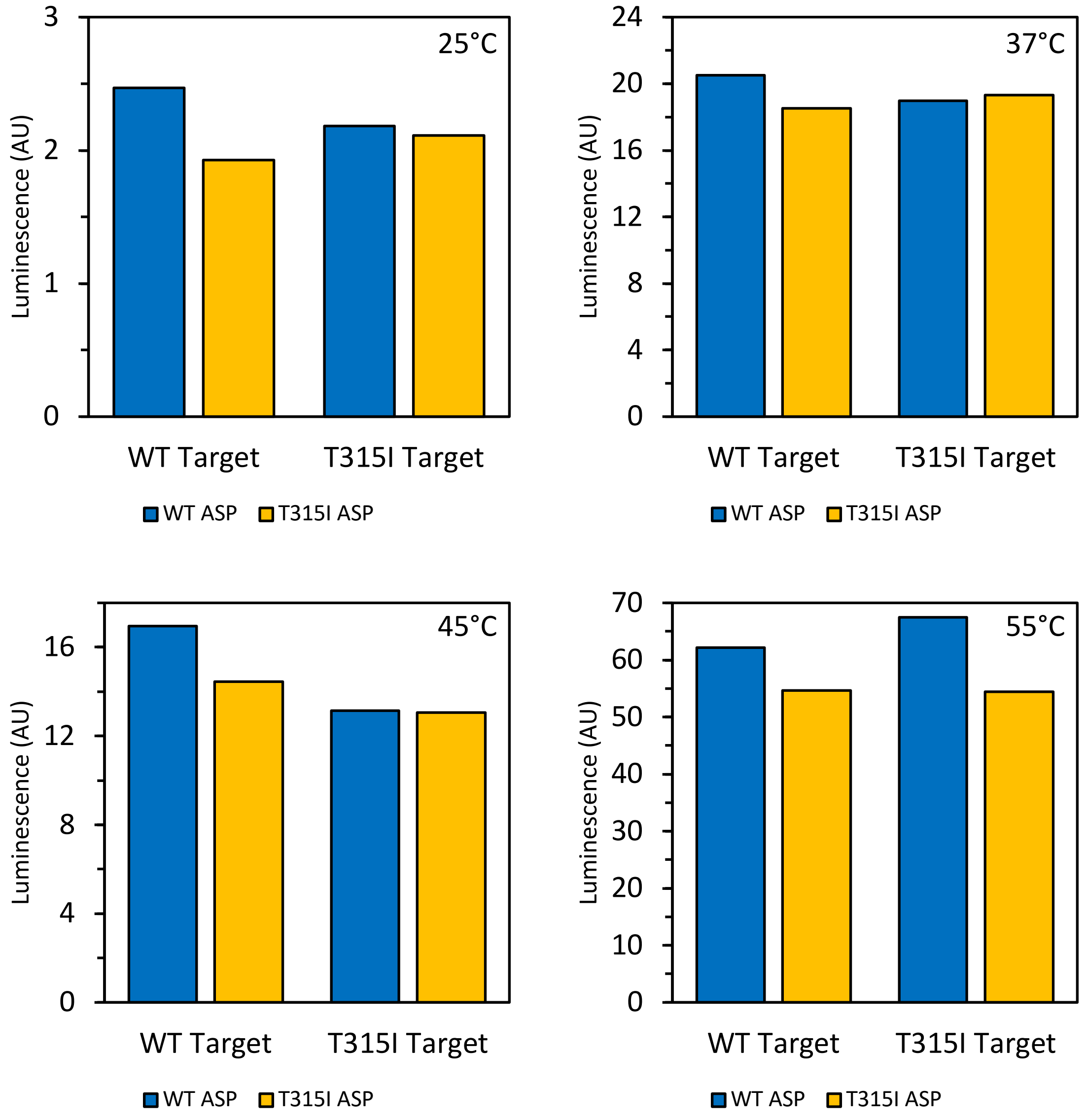

Figure S6. Specificity for matches over mismatches with *BCR-ABL1* 488mer RNA targets while varying incubation temperature. 100 nM target, 1 μM ASP, 20 μM ARNs (all four), Maxima H Minus RT. Incubated 30 min at varied temperatures as indicated. Increasing temperature increased signal but did not improve specificity.

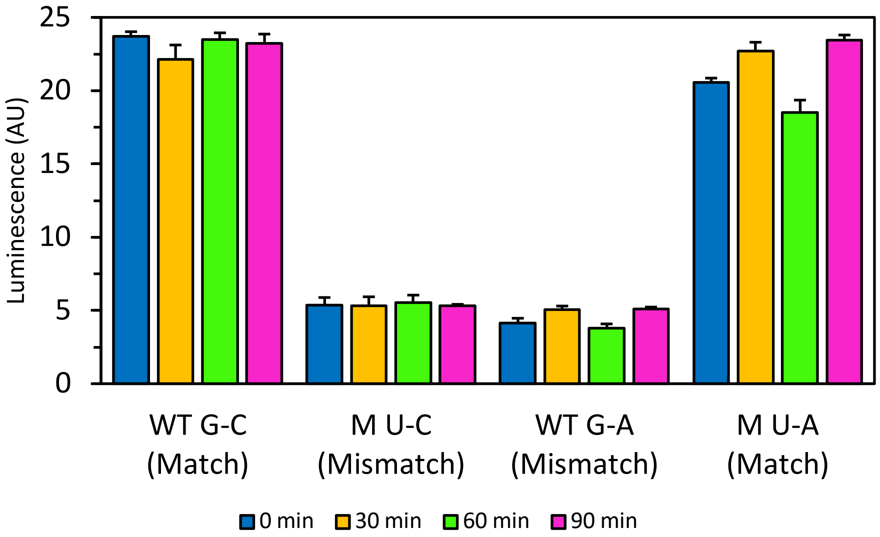

Figure S7. Specificity for matches over mismatches with *JAK2* 500mer RNA targets while varying pre-incubation time at room temperature. 100 nM target, 1 µM ASP incubated in reaction buffer at RT. 20 µM ARNs (all four), Maxima H Minus RT incubated 30 min at 37°C. Increasing pre-incubation time does not appear to affect specificity.

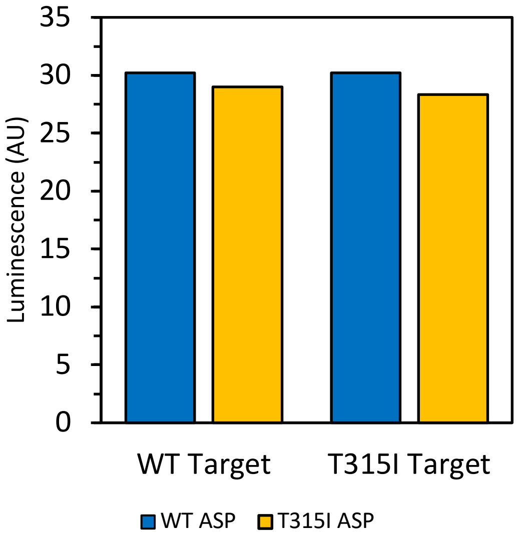

Figure S8. Specificity for matches over mismatches with *BCR-ABL1* 488nt RNA targets with an annealing step prior to the reaction. 100 nM target, 1 µM ASP were annealed in reaction buffer by incubating at 80°C for 2 min, then slowly cooling to 4°C at a rate of 1°/s. After annealing, 20 µM ARNs (all four) were added, followed by Maxima H Minus RT. Incubated 30 min at 37°C. No specificity was attained with the added annealing step.

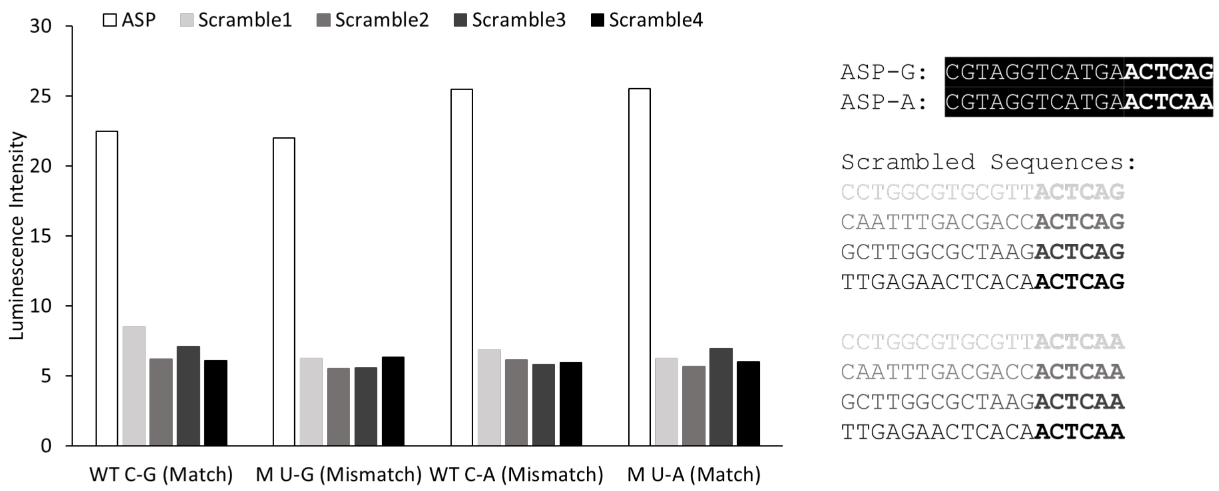

Figure S9. To test the possibility of accidental homology of ASP throughout the *BCR-ABL1* target, ASP sequences were randomized, though the first six nucleotides from the 3′ end were conserved. 100 nM target, 1 µM ASP (all four), 20 µM ARNs, Maxima H Minus RT. Incubated 30 min at 37°C. None of the randomized primers gave any significant signal above background luminescence.

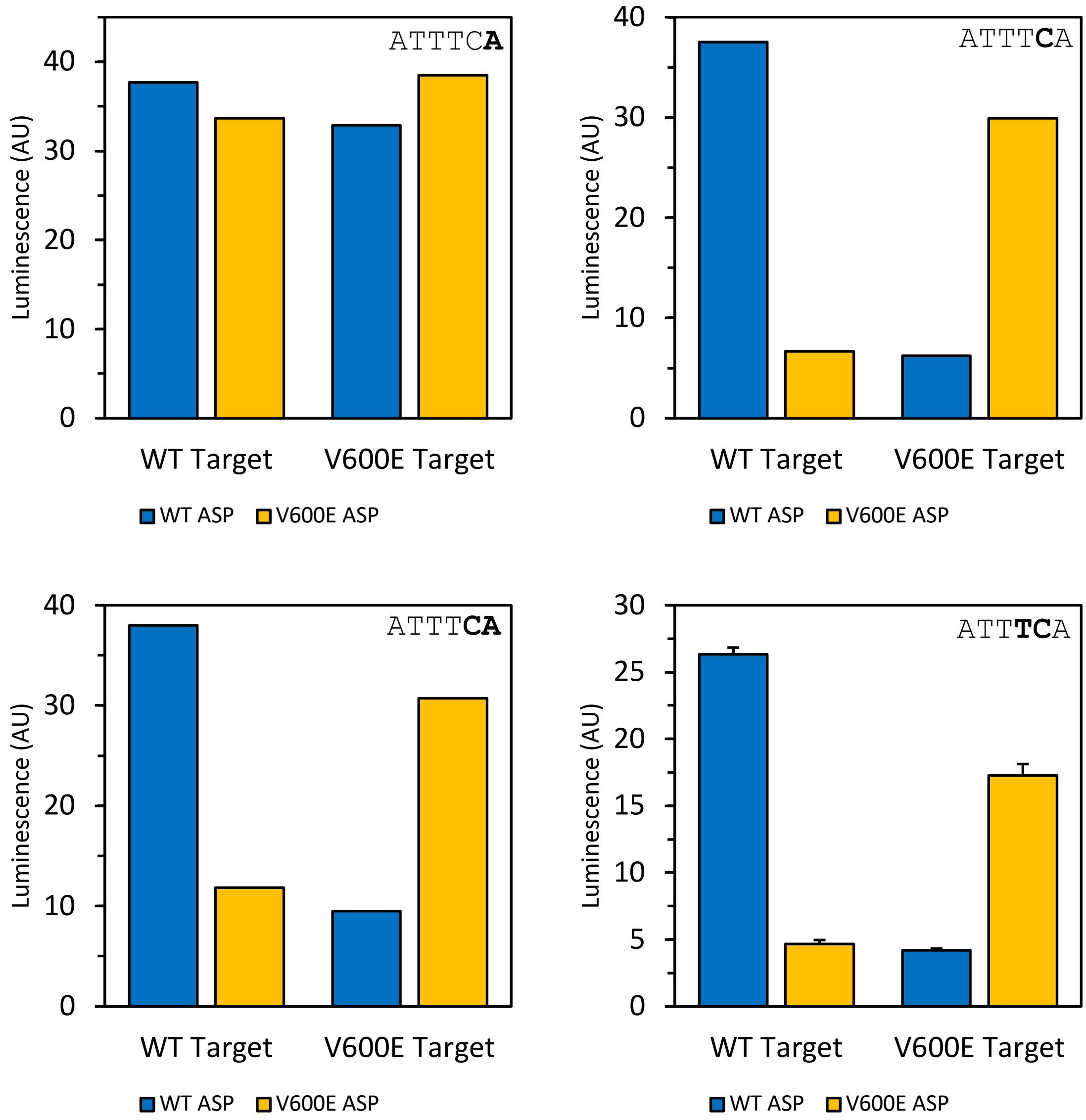

Figure S10. Testing different LNA-containing ASPs with *BRAF* 500nt RNA targets. The index in the right-hand corner indicates the position of the LNA nucleotides (in bold) in the ASP (the last six nucleotides at the 3′ end are shown). 100 nM target, 1 μM ASP, 20 μM ARNs, Maxima H Minus RT, incubated 30 min at 37°C. Including an LNA nucleotide at the 3′ end reduced specificity; however, placing the LNA 1 nucleotide away from 3′ end increased specificity relative to normal ASP.

A

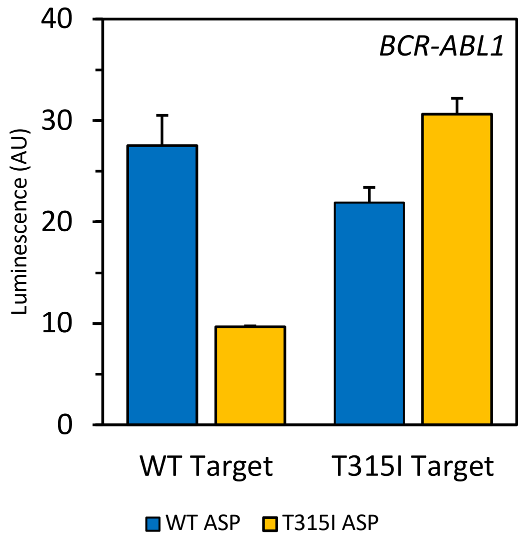

B

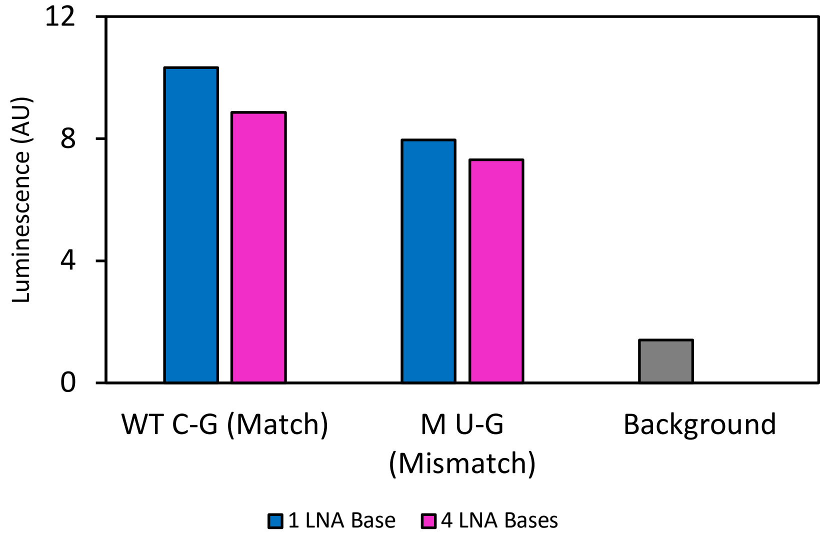

Figure S11. Testing the effects of LNA-containing ASPs on problematic RNA sequences (*BCR-ABL1* and *HBB*/beta-globin). **A.** Improved specificity for matches over mismatches with LNA-containing ASPs for *BCR-ABL1* and *HBB*/beta-globin alleles. The ASP contained 1 LNA-modified residue at the second from the 3′ end. 100 nM RNA target, 1 µM ASP, 20 µM ARNs (A, T, G), 20 µM dCTP, Maxima H Minus RT. Orange: *BCR-ABL1*, incubated 30 min at 37°C; Red: *HBB*, incubated 1 hr at 37°C. **B.** Under same conditions, ASP containing 3 additional LNA nucleotides (including the LNA nucleotide at the second from the 3′ end) did not improve specificity for *BCR-ABL1*.

A

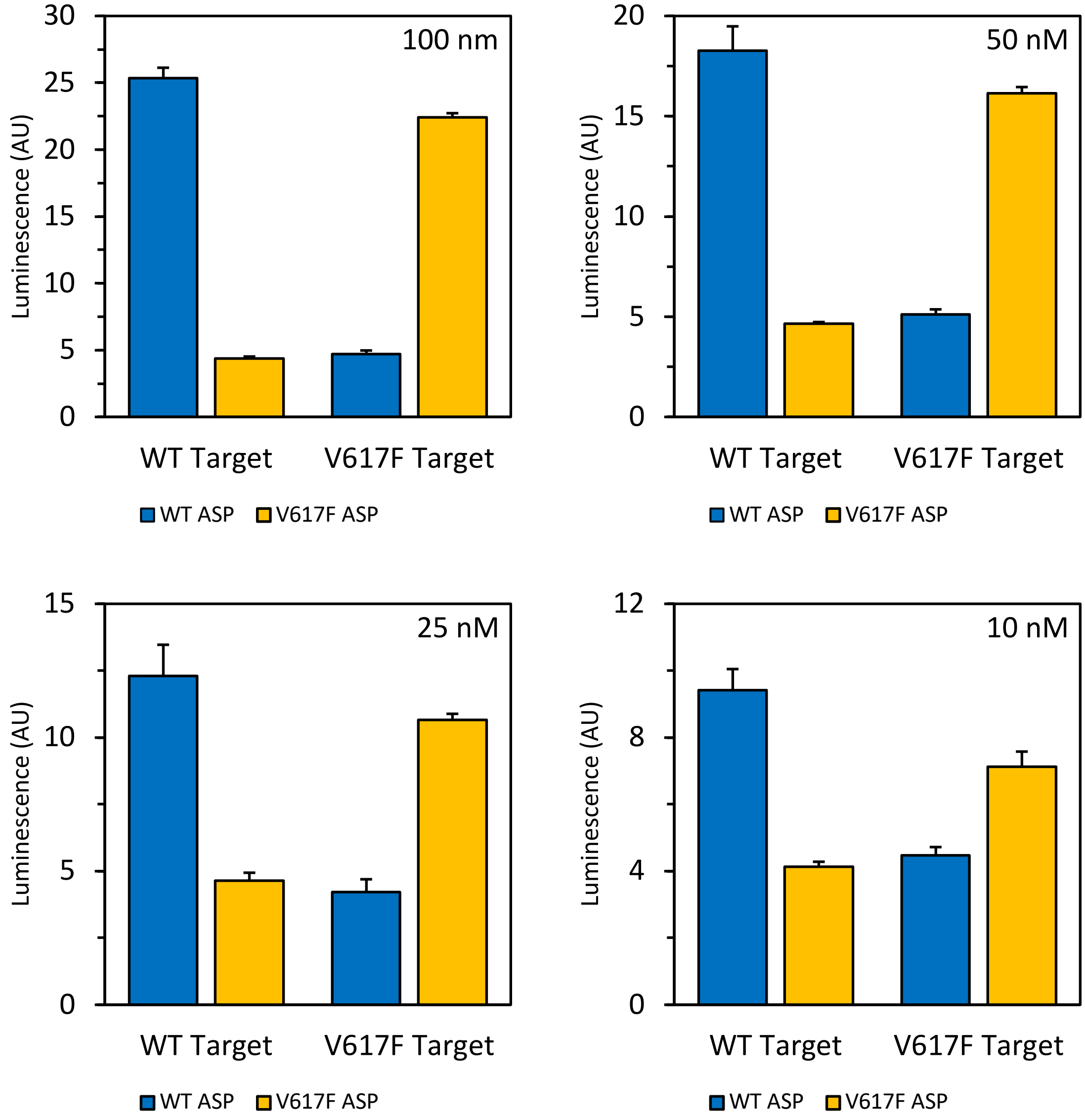

30
25
20
15
10
5
0
Luminescence (AU)
100 nm
WT Target
V617F Target
WT ASP
V617F ASP
20
15
10
5
0
Luminescence (AU)
50 nM
WT Target
V617F Target
WT ASP
V617F ASP
15
10
5
0
Luminescence (AU)
25 nM
WT Target
V617F Target
WT ASP
V617F ASP
12
8
4
0
Luminescence (AU)
10 nM
WT Target
V617F Target
WT ASP
V617F ASP

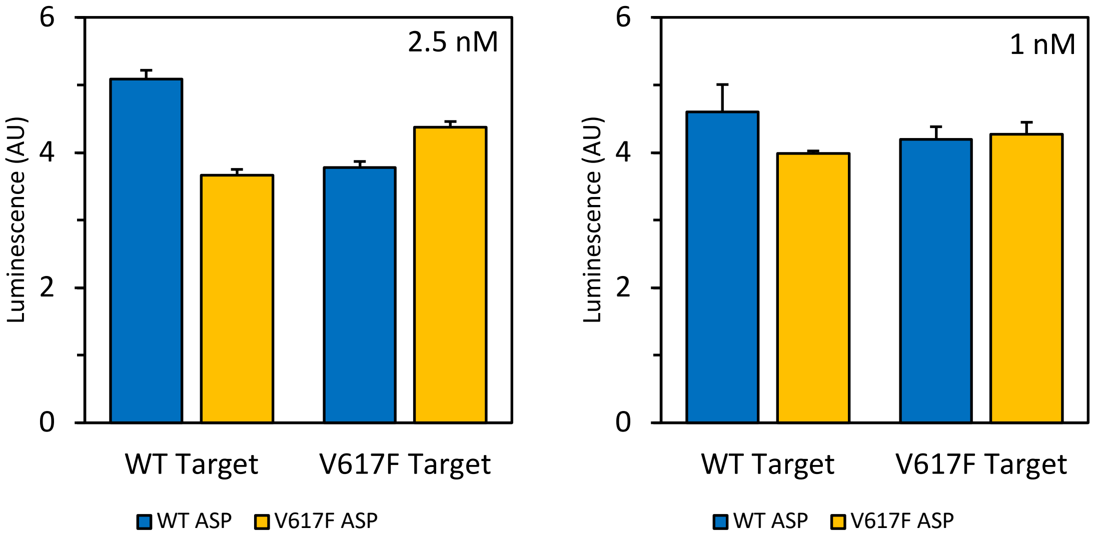

B

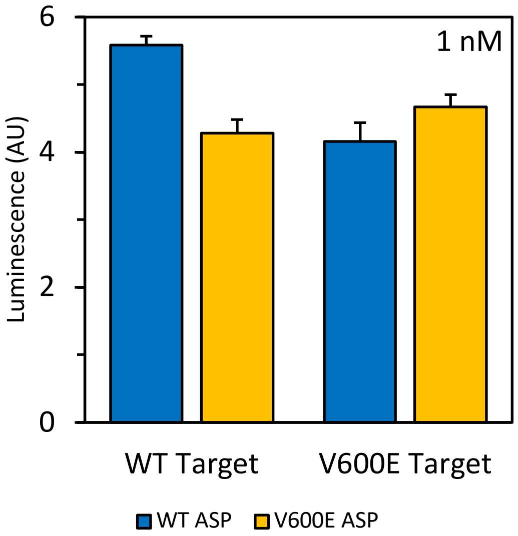

Figure S12. Determining limit of detection for RNA targets. **A.** Varying concentration of *JAK2* 500nt RNA target with 1 µM ASP, 20 µM ARNs, Maxima H Minus RT, incubated for 30 min at 37°C. Matches could be discriminated from mismatches at target concentrations of 100 nM (2 pmol), 50 nM (1 pmol), 25 nM (0.5 pmol), 10 nM (0.2 pmol), and 2.5 nM (50 fmol). Limit of detection determined to be 2.5 nM for *JAK2* 500nt RNA targets. **B.** Specificity with *BRAF* 500nt RNA targets at 1 nM target concentrations. Matches could be discriminated from mismatches at target concentration of 1 nM (25 fmol). Limit of detection determined to be 1 nM for *BRAF* 500mer RNA targets.

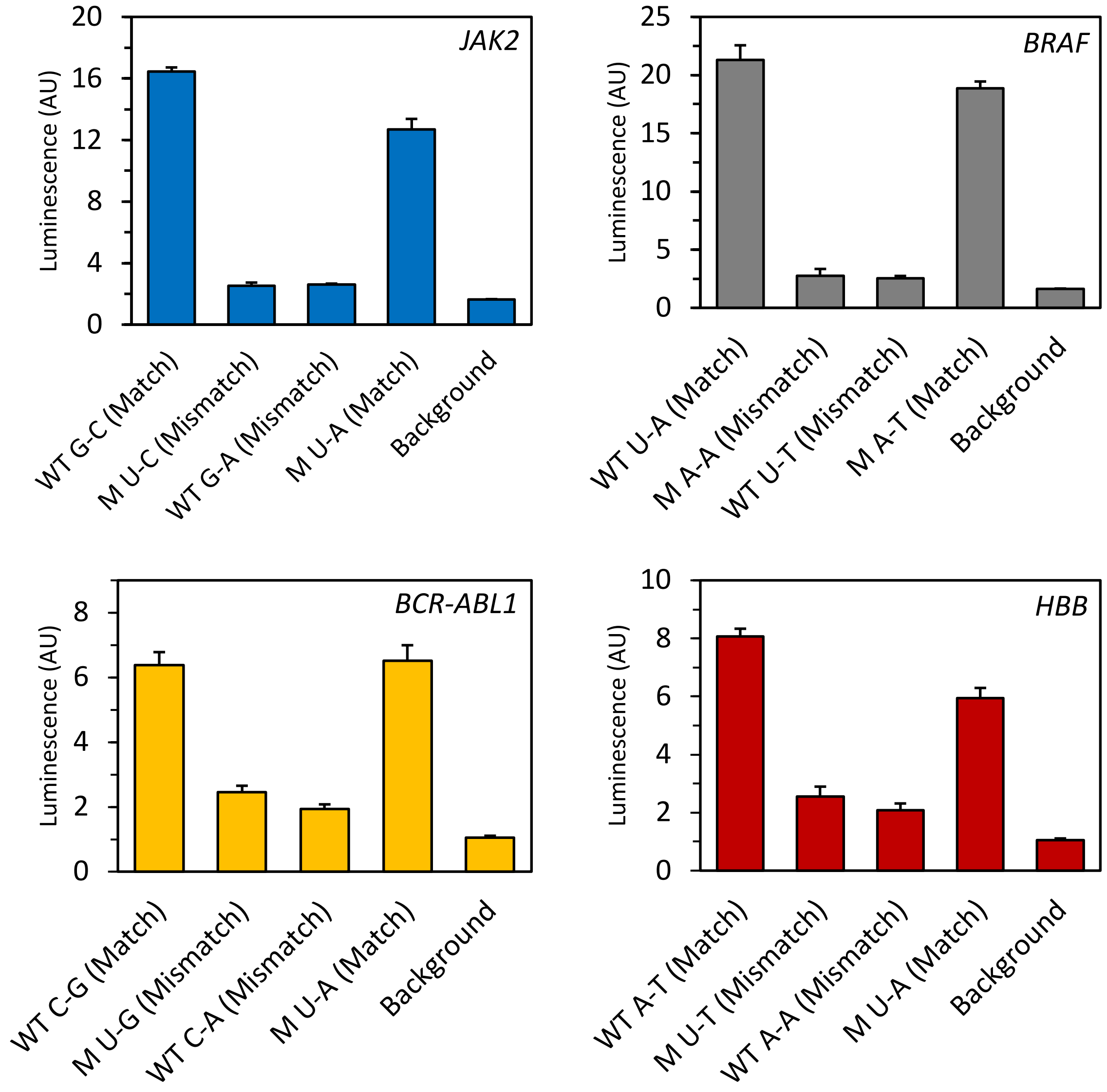

Figure S13. Selectivity for matches over mismatches with all four alleles: 100 nM ~500nt RNA target, 1 µM ASP, 20 µM ARNs (A, T, G), 20 µM dCTP, Maxima H Minus RT. Incubated for 30 min at 37°C, except for the shorter *HBB* targets which were incubated for 1 hr. After subtracting background luminescence (Figure 2 in main text), signal increases for matches over mismatches were as follows: *JAK2* (14-fold), *BRAF* (18-fold), *BCR-ABL1* (4.3-fold), *HBB*/beta-globin (4.7-fold).

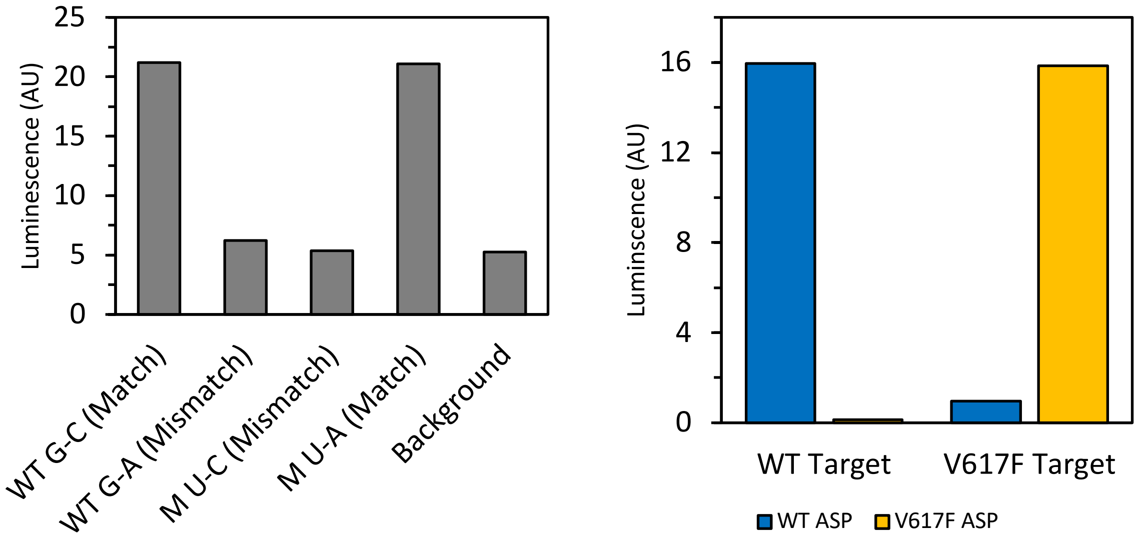

Figure S14. Double-extension experiment with *JAK2* 500nt RNA targets. Reaction tubes containing 100 nM *JAK2* mRNA mimics, 1 µM ASP, 20 µM ARNs (A, T, G), 20 µM dCTP, and Maxima H Minus RT were incubated at 37°C for 30 min. After 30 min, RNAse-H was added to each mixture, which was then incubated at 37°C for 15 min. After this time, 0.5 µL Klenow exo- and 2.0 µL 10 µM *JAK2* Forward Primer were added to each reaction vessel, which was then incubated at 37°C for an additional 30 min. After subtracting background luminescence, signals were 29-fold greater for matches over mismatches.

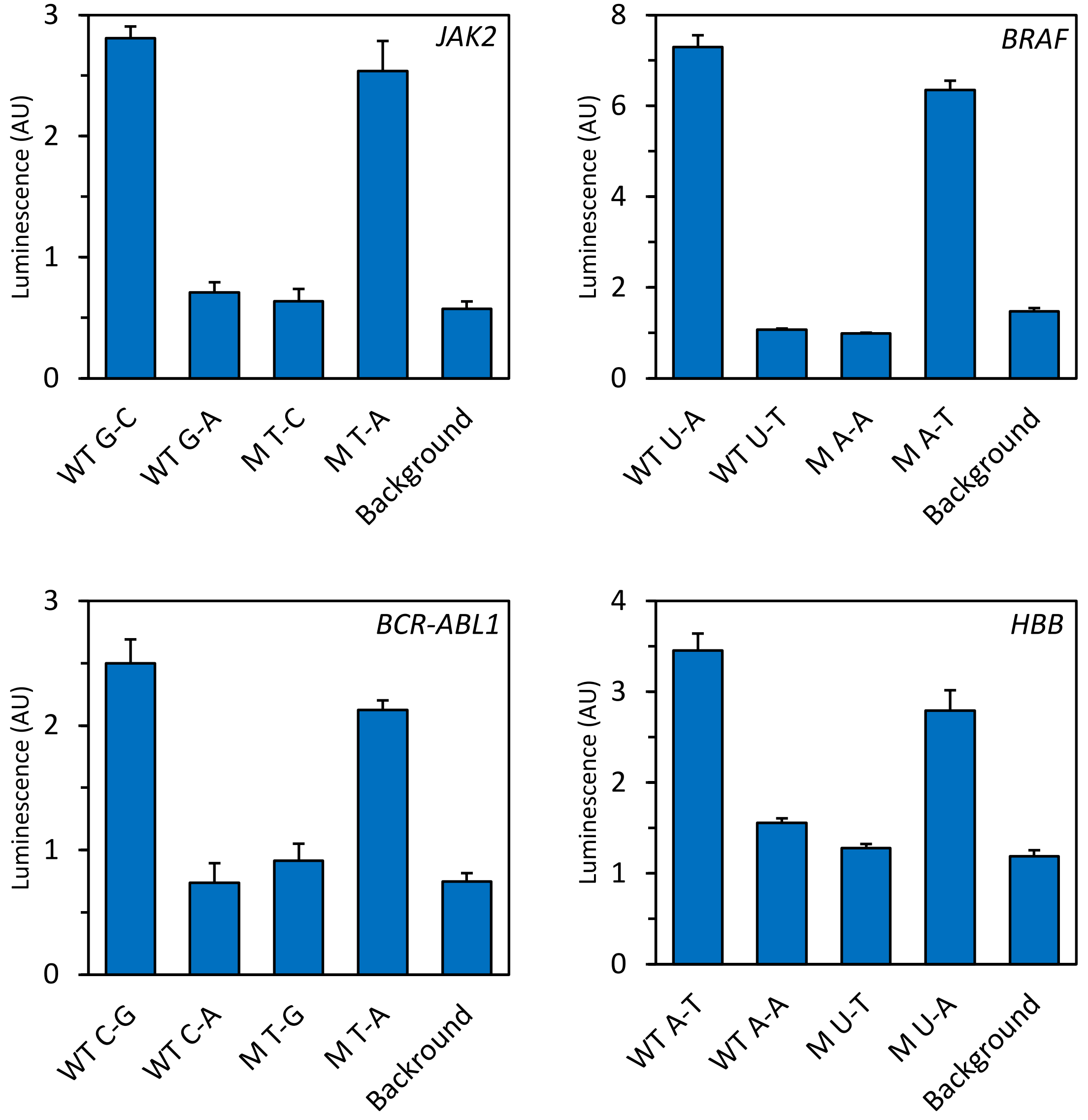

Figure S15. Specificity of wild-type (WT) vs mutant (M) mRNA mimics amid total cellular RNA isolated from a human blood sample. 100 nM ~500nt RNA target (except for *HBB*), 112.5 ng total cellular RNA, 1 µM ASP, 20 µM ARNs (A, T, G), 20 µM dCTP, Maxima H Minus RT. Incubated for 30 min at 37°C (1 hr for *HBB*). After subtracting background luminescence, signals for matches between target and ASP were as follows: *JAK2* (22-fold), *BRAF* (no signal over background), *BCR-ABL1* (20-fold), *HBB* (8.5-fold). Background luminescence was measured by running a parallel experiment without ASP. (See Figure 3 in main text).

Sequence Data

LNA monomers are shown in bold.

Strand Name	Length	Sequence (5′→3′)
JAK2 WT Target-G	50	ACA AGC ATT TGG TTT TAA ATT ATG GAG TAT GTG TCT GTG GAG ACG AGA AT
JAK2 Mut Target-T	50	ACA AGC ATT TGG TTT TAA ATT ATG GAG TAT GTT TCT GTG GAG ACG AGA AT
JAK2 WT ASP-C	18	ATT CTC GTC TCC ACA GAC
JAK2 Mut ASP-A	18	ATT CTC GTC TCC ACA GAT
BRAF WT Target-T	50	AGT AAA AAT AGG TGA TTT TGG TCT AGC TAC AGT GAA ATC TCG ATG GAG TG
BRAF Mut Target-A	50	AGT AAA AAT AGG TGA TTT TGG TCT AGC TAC AGA GAA ATC TCG ATG GAG TG
BRAF WT ASP-A	18	CAC TCC ATC GAG ATT TCA
BRAF Mut ASP-T	18	CAC TCC ATC GAG ATT TCT
BCR-ABL1 WT Target-C	50	CTG CAC CCG GGA GCC CCC GTT CTA TAT CAT CAC TGA GTT CAT GAC CTA CG
BCR-ABL1 Mut Target-T	50	CTG CAC CCG GGA GCC CCC GTT CTA TAT CAT CAT TGA GTT CAT GAC CTA CG
BCR-ABL1 WT ASP-G	18	CGT AGG TCA TGA ACT CAG
BCR-ABL1 Mut ASP-A	18	CGT AGG TCA TGA ACT CAA
HBB WT Target-A	50	TCA AAC AGA CAC CAT GGT GCA TCT GAC TCC TGA GGA GAA GTC TGC CGT TA
HBB Mut Target-T	50	TCA AAC AGA CAC CAT GGT GCA TCT GAC TCC TGT GGA GAA GTC TGC CGT TA
HBB WT ASP-T	18	TAA CGG CAG ACT TCT CCT
HBB Mut ASP-A	18	TAA CGG CAG ACT TCT CCA
JAK2 Fwd PCR Primer	21	TGA AGA GTA CAA CCT CAG TGG
JAK2 Rev PCR Primer	21	TGA ACC AGA ATA TTC TCG TCT
JAK2 Fwd T7 PCR Primer	41	TAA TAC GAC TCA CTA TAG GGT GAA GAG TAC AAC CTC AGT GG

BRAF Rev PCR Primer	18	5′-Biotin-TTC TGA TGA CTT CTG GTG
BRAF Fwd T7 PCR Primer	38	TAA TAC GAC TCA CTA TAG GGA CAA AGA ATT GGA TCT GG
BCR-ABL1 Fwd PCR Primer	23	CGT AGG TCA TGA ACT CAG TGA TG
BCR-ABL1 WT Rev Primer	23	CGT AGG TCA TGA ACT CAG TGA TG
BCR-ABL1 Mut Rev Primer	23	CGT AGG TCA TGA ACT CAA TGA TG
BCR-ABL1 Fwd T7 PCR Primer	38	TAA TAC GAC TCA CTA TAG GGC AGA GGT CCA TCT CGC TG
HBB WT T7 Template	107	TAA TAC GAC TCA CTA TAG GGA CAT TTG CTT CTG ACA CAA CTG TGT TCA CTA GCA ACC TCA AAC AGA CAC CAT GGT GCA TCT GAC TCC TGA GGA GAA GTC TGC CGT TA
HBB Mut T7 Template	107	TAA TAC GAC TCA CTA TAG GGA CAT TTG CTT CTG ACA CAA CTG TGT TCA CTA GCA ACC TCA AAC AGA CAC CAT GGT GCA TCT GAC TCC TGT GGA GAA GTC TGC CGT TA
HBB Fwd Primer	19	TAA TAC GAC TCA CTA TAG G
HBB Rev Primer	17	TAA CGG CAG ACT TCT CC
BCR-ABL1 ASP-G Random1	18	CGC GTC TCT GTG GCT CAG
BCR-ABL1 ASP-G Random2	18	GCC CAT CAT CTA CAT CAG
BCR-ABL1 ASP-G Random3	18	TCC AGT TCA TGT ATT CAG
BCR-ABL1 ASP-G Random4	18	GTA AGC TCG TAC TGT CAG
BCR-ABL1 ASP-A Random1	18	CGC GTC TCT GTG GCT CAA
BCR-ABL1 ASP-A Random2	18	GCC CAT CAT CTA CAT CAA
BCR-ABL1 ASP-A Random3	18	TCC AGT TCA TGT ATT CAA
BCR-ABL1 ASP-A Random4	18	GTA AGC TCG TAC TGT CAA
BCR-ABL1 ASP-T	18	CGT AGG TCA TGA ACT CAT
BCR-ABL1 ASP-C	18	CGT AGG TCA TGA ACT CAC
BRAF WT LNA-C ASP-A	18	CAC TCC ATC GAG ATT T**C**A
BRAF Mut LNA-C ASP-T	18	CAC TCC ATC GAG ATT T**C**T

BRAF WT LNA-TC ASP-A	18	CAC TCC ATC GAG ATT **TC**A
BRAF Mut LNA-TC ASP-T	18	CAC TCC ATC GAG ATT **TC**T
BRAF WT LNA-A ASP-A	18	CAC TCC ATC GAG ATT TC**A**
BRAF Mut LNA-T ASP-T	18	CAC TCC ATC GAG ATT TC**T**
BRAF WT LNA-CA ASP-A	18	CAC TCC ATC GAG ATT T**CA**
BRAF Mut LNA-CT ASP-T	18	CAC TCC ATC GAG ATT T**CT**
BCR-ABL1 WT LNA-A ASP-G	18	CGT AGG TCA TGA ACT C**A**G
BCR-ABL1 Mut LNA-A ASP-A	18	CGT AGG TCA TGA ACT C**A**A
HBB WT LNA-C ASP-T	18	TAA CGG CAG ACT TCT C**C**T
HBB Mut LNA-C ASP-A	18	TAA CGG CAG ACT TCT C**C**A
BCR-ABL1 WT LNA-4 ASP-G	18	CGT A**G**G TC**A** TGA **A**CT C**A**G
BCR-ABL1 Mut LNA-4 ASP-A	18	CGT A**G**G TC**A** TGA **A**CT C**A**A
JAK2 WT LNA-GA ASP-C	18	ATT CTC GTC TCC ACA **GA**C
JAK2 Mut LNA-GA ASP-A	18	ATT CTC GTC TCC ACA **GA**A
BCR-ABL1 WT LNA-CA ASP-G	18	CGT AGG TCA TGA ACT **CA**G
BCR-ABL1 Mut LNA-CA ASP-A	18	CGT AGG TCA TGA ACT **CA**A
HBB WT LNA-CC ASP-T	18	TAA CGG CAG ACT TCT **CC**T
HBB WT LNA-CC ASP-A	18	TAA CGG CAG ACT TCT **CC**A

References

1. Ji, D., Mohsen, M. G., Harcourt, E. M. & Kool, E. T. ATP-releasing nucleotides: Linking DNA synthesis to luciferase signaling. *Angew. Chemie - Int. Ed.* (2016). doi:10.1002/anie.201509131
2. Brady, D. C. *et al.* Copper is required for oncogenic BRAF signalling and tumorigenesis. *Nature* (2014). doi:10.1038/nature13180
3. Johannessen, C. M. *et al.* COT drives resistance to RAF inhibition through MAP kinase pathway reactivation. *Nature* (2010). doi:10.1038/nature09627
4. Martz, C. A. *et al.* Systematic identification of signaling pathways with potential to confer anticancer drug resistance. *Sci. Signal.* (2014). doi:10.1126/scisignal.aaa1877
5. He, Y. *et al.* The coiled-coil domain and Tyr177 of bcr are required to induce a murine chronic myelogenous leukemia-like disease by bcr/abl. *Blood* (2002). doi:10.1182/blood.V99.8.2957
